# 

**Published:** 1893-01-21

**Authors:** 


					?^ IT
w w WITH EXTRA NURSING SUPPLEMENT.
TWOPENCE. 00* lit?'If )1^^\ Per Annum. 8s. 6d., post free.
Monthly Farts, 6d.; post free, 8d.
?.5o'9e IWOPENCE.
Vol. XIII.?Jan. 21st, 1893.
thfi OamamI t> ..   - a.
tnj?t ttw General Poet Office ai a Newspaper for Mk> ? Ditto per Annnmj 8s., post free.
ln the United Kingdom and Abroad.]
Vol. XUX] Saturday,
Jantjaey 21st, IS 93.
[Twopence.

				

